# Novel splicing in IGFN1 intron 15 and role of stable G-quadruplex in the regulation of splicing in renal cell carcinoma

**DOI:** 10.1371/journal.pone.0205660

**Published:** 2018-10-18

**Authors:** Shiv Prakash Verma, Parimal Das

**Affiliations:** Centre for Genetic Disorders, Institute of Science, Banaras Hindu University, Varanasi, INDIA; Iowa State University, UNITED STATES

## Abstract

The IGFN1 (Immunoglobulin-Like And Fibronectin Type III Domain Containing 1) gene has a role in skeletal muscle function and is also involved in metastatic breast cancer, and the isoforms with three N-terminal globular domains are sufficient for its function in skeletal muscle. Two novel splicing isoforms of IGFN1 have been identified in renal cell carcinoma (RCC), one with 5’exon extension and an isoform with a novel exon. The role of G-quadruplex, a non-B DNA, was explored for the splicing alteration of IGFN1 in RCC. G-quadruplexes are the secondary structures acquired by stacking of G-quartets by Hoogsteen hydrogen bonding in DNA and RNA. IGFN1 has intronic potential G-quadruplex forming sequence (PQS) folding into G-quadruplex and is studied for its involvement in aberrant splicing. A PQS in the intron 15 of IGFN1 gene was observed in our *in silico* analysis by QGRS mapper and non BdB web servers. We observed PQS folds into stable G-quadruplex structure in gel shift assay and circular dichroism (CD) spectroscopy in the presence of G-quadruplex stabilizing agents Pyridostatin (PDS) and KCl, respectively. G-quadruplex formation site with single base resolution was mapped by Sanger sequencing of the plasmid constructs harbouring the cloned PQS and its mutant. This stable G-quadruplex inhibits reverse transcriptase and taq polymerase in reverse transcriptase & PCR stop assays. PDS changes the different splicing isoforms of IGFN1 in UOK146 cell line, displaying involvement of intronic G-quadruplex in IGFN1 splicing. These results lead us to propose that a stable G-quadruplex structure is formed in IGFN1 intron and a reason behind IGFN1 aberrant splicing which could be targeted for therapeutic intervention.

## Introduction

IGFN1 is specifically expressed in skeletal muscle and has sequence and structural homology to myosin binding protein-C fast and slow-type skeletal muscle isoforms. During muscle denervation IGFN1 is substantially upregulated leading to the down-regulation of protein synthesis via eEF1A interaction [1]. In Chinese population, IGFN1 is associated with susceptibility to Primary retroperitoneal liposarcoma [2].

IGFN1 along with KY and FLNC is the part of a Z-band associated protein complex providing structural support to the skeletal muscle [3]. IGFN1 is frequently mutated in metastatic breast cancer as compared to early breast cancer [4]. Expression and role of different isoforms of recombinant fragment of IGFN1 have shown that three N-terminal globular domains, common to at least five IGFN1 variants, are sufficient for Z-band targeting [3]. Alternative splicing regulates the proteomic diversity and apart from different splicing factors, RNA sequences with Non-B DNA structures are also involved in the isoform specific gene expression. Non-B DNA is the non canonical form of DNA involved in almost all basic biological process e.g. replication, transcription, translation etc. These types of structure include hairpins/cruciforms, triplexes (H-DNA), Z-DNA, tetraplex, sticky DNA, slipped-DNA and G-quadruplex [5–7]. Among all these G-quadruplexes are studied extensively for its role in different types of diseases such as cancer and neurodegenerative disorders [8–13]. G-quadruplexes are a non-B form of nucleic acid secondary structures formed by the interaction of planar G-quartet building blocks through a cyclic Hoogsten hydrogen-bonding arrangement of four guanines [6] and are involved in all cancer hallmarks [14–16]. Bioinformatic analysis shows that 16654 genes in the human RefSeq database has Potential for G4 DNA formation (G4P) and tumor suppressor genes have very low G4P and protooncogenes have very high G4P [17]. At DNA level G-quadruplexes are involved in BCL2 major breakpoint region t(14;18) translocation follicular lymphoma [18], HOX11 gene in t(10;14) translocation in T-cell leukemia [19], cMyc translocation and hypermutation [20], telomere replication.

At RNA level G-quadruplexes regulate transcription and posttranscriptional modification causing pathogenesis in many diseases. G-quadruplex in intron 3 of TP53 gene has been shown to regulate alternative splicing of p53 messenger RNA [21–24], and it has been demonstrated that G4 polymorphisms in haplotypes of the WT TP53 allele have an impact on Li-Fraumeni/Li-Fraumeni-like syndrome (LFS/LFL) penetrance in germline TP53 mutation carriers [24]. Interaction between a G-quadruplex structure located downstream from the p53 cleavage site and hnRNP H/F is critical for p53 expression and contributes to p53-mediated apoptosis [25]. Hexanucleotide (GGGGCC) repeat in C9ORF72 is the most frequent known cause of amyotrophic lateral sclerosis (ALS) and frontotemporal dementia (FTD). This expanded transcript adopts G-quadruplex structures and interacts with splicing factor hnRNP H protein. This expansion sequesters hnRNP H and is a significant contributor to neurodegeneration in ALS/FTD [12]. Another ribonucleoprotein Nucleolin also interacts with the aborted repeat transcripts and provides the basis for a mechanistic model for repeat-associated neurodegenerative diseases [13].

In the present study the role of G-quadruplex in splicing of IGFN1 is studied in Xp11.2 tRCC. We investigated the possibility of folding of PQS into a G-quadruplex structure by different assays e.g. Gel shift assay, CD spectroscopy, PCR stop assay, transcriptional inhibition and pyridostatin effect on splicing. A biologically stable G-quadruplex was formed in the intronic sequence of IGFN1, which regulates splicing and hence different isoform level.

## Results

### 1. Splicing analysis of UOK146 cell line and IGFN1 aberrant splicing

**Fig 1:**
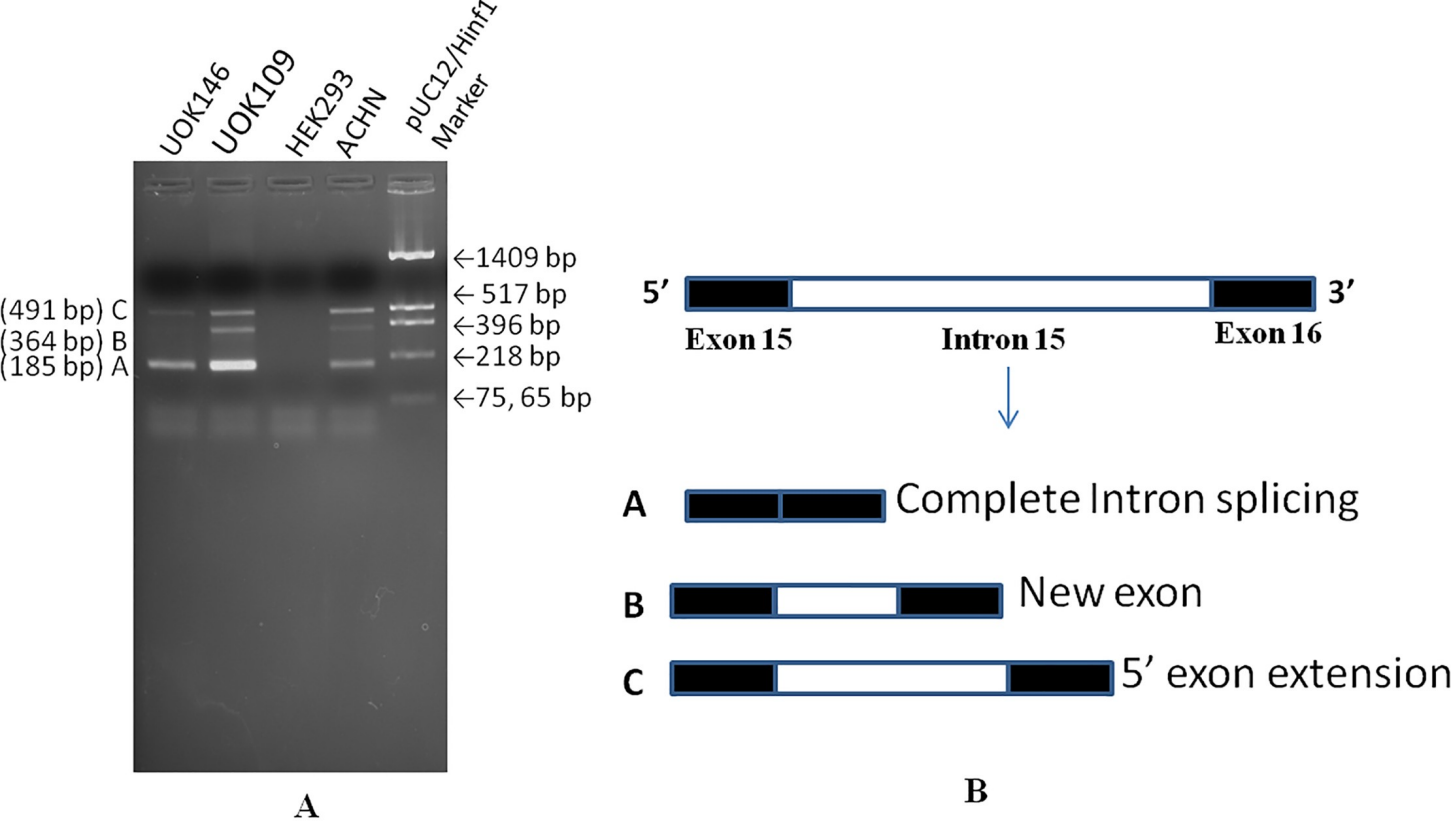
Analysis for novel splicing in UOK146 cell line. (A) Validation of novel splicing pattern in intron 15 of IGFN1 by RT-PCR by sense primer from exon 15 and antisense primer from exon 16, (B) Map of the novel spliced isoforms after the Sanger sequencing confirmation. Intron 15 is completely spliced in isoform A, a novel exon was identified in isoform B and 5’ exon extension was observed in isoform C.

Novel splicing in IGFN1 was identified, validated and confirmed by RT-PCR (Fig 1A) and Sanger sequencing. RT-PCR from UOK146 and other RCC cell lines e.g UOK109 and ACHN identified three isoforms (amplicons) named as A, B and C with increasing mol. wt. respectively. In UOK146 only amplicon A & C were present whereas amplicon B along with A & C were present in other RCC cell lines e.g. UOK109 and ACHN. Sequence analysis of all these three amplicon showed two novel splicing events (Fig 1B). Amplicon A with lowest size (185bp) showed the complete intron 15 splicing from the isoform as normally reported in reference databases. Amplicon B having the size of 364 bp has a novel exon matching the sequences from intron 15. Amplicon C of 491 bp has the 5’exon extension covering the intron 15 sequences.

### 2. G-rich sequence with PQS is present in IGFN1 Intron 15

**Fig 2:**
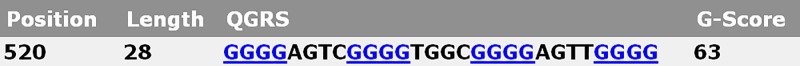
G-quadruplex prediction. QGRS mapper prediction of G-quadruplex for the IGFN1 intron15 displayed a PQS with score 63.

PQS prediction in intron 15 of IGFN1 mRNA (NM001164586.1) was performed by a web based server QGRS mapper (www.bioinformatics.ramapo.edu/QGRS/analyze.php) and a PQS was identified. This sequence was further analysed by a non-B DNA structure analysis database, non-B DB (https://nonb-abcc.ncifcrf.gov/apps/nBMST/default/), same sequence was predicted as PQS (Fig 2).

### 3. Gel shift assay

**Fig 3:**
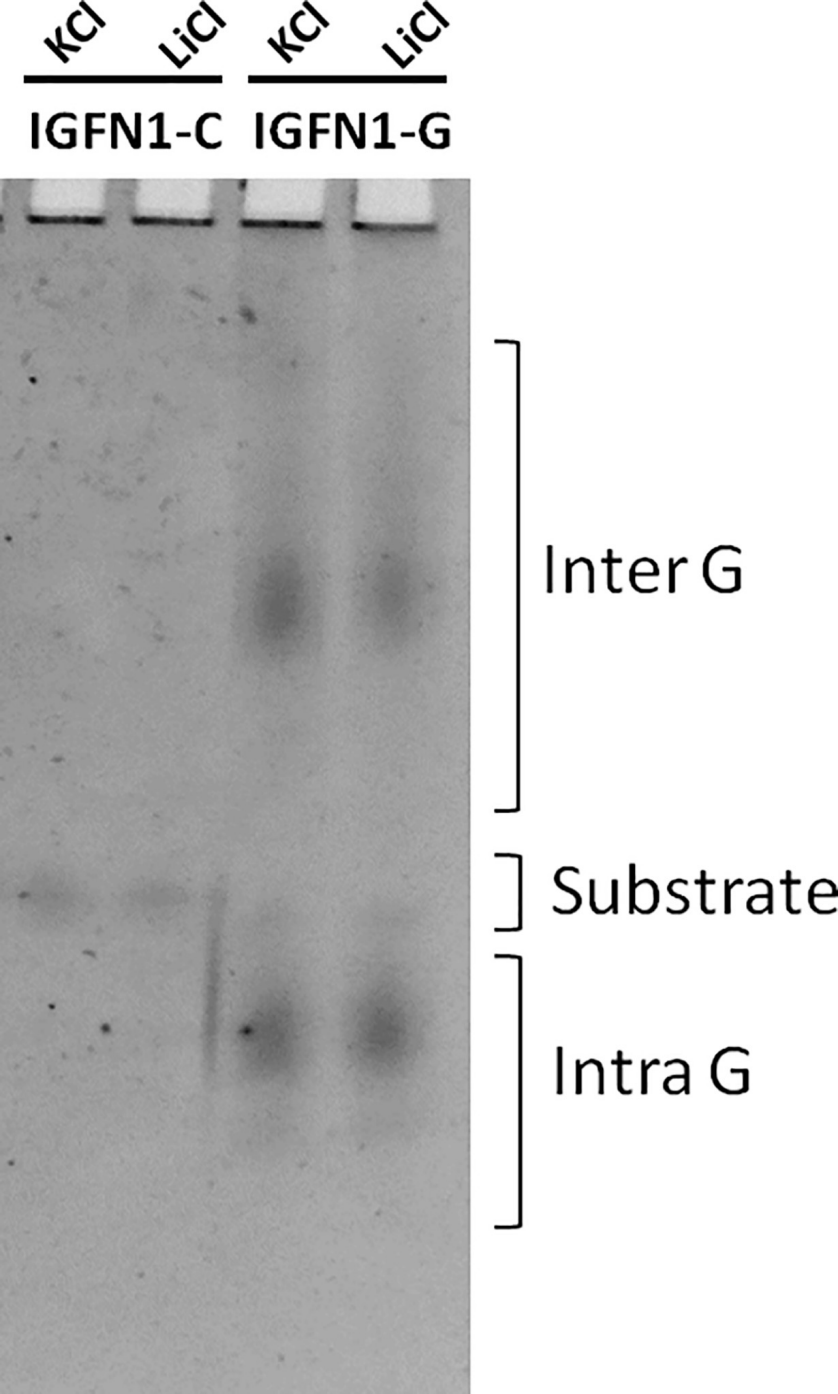
Gel shift assay by native PAGE. IGFN1-G and IGFN1-C incubated with 100 mM LiCl and KCl and resolved on 15% native PAGE, intra and intermolecular G-quadruplex structures are mentioned.

G-quadruplex secondary structure formation in IGFN1 intron 15 was studied by mobility shift assay of oligos bearing PQS. Secondary structures of IGFN1-G oligomer with increased and decreased mobility was formed as displayed in gel shift assay. IGFN1-G oligo forms intra and inter molecular G-quadruplex structures in presence of LiCl and KCl has, displaying IGFN1-G inherent property to form the intra and inter molecular G-quadruplex structures. Whereas IGFN1-C(reverse complement of IGFN1-G) is unable to form these secondary structures because guanosine are replaced by cytosine (Fig 3).

**IGFN1-G:** 5’-ATGGGGGTGGGGGGGAGTCGGGGTGGCGGGGAGT-3’

**IGFN1-C:** 5’-ACTCCCCGCCACCCCGACTCCCCCCCACCCCCAT-3’

### 4. KCl stabilizes G-quadruplex secondary structures formed IGFN1

**Fig 4:**
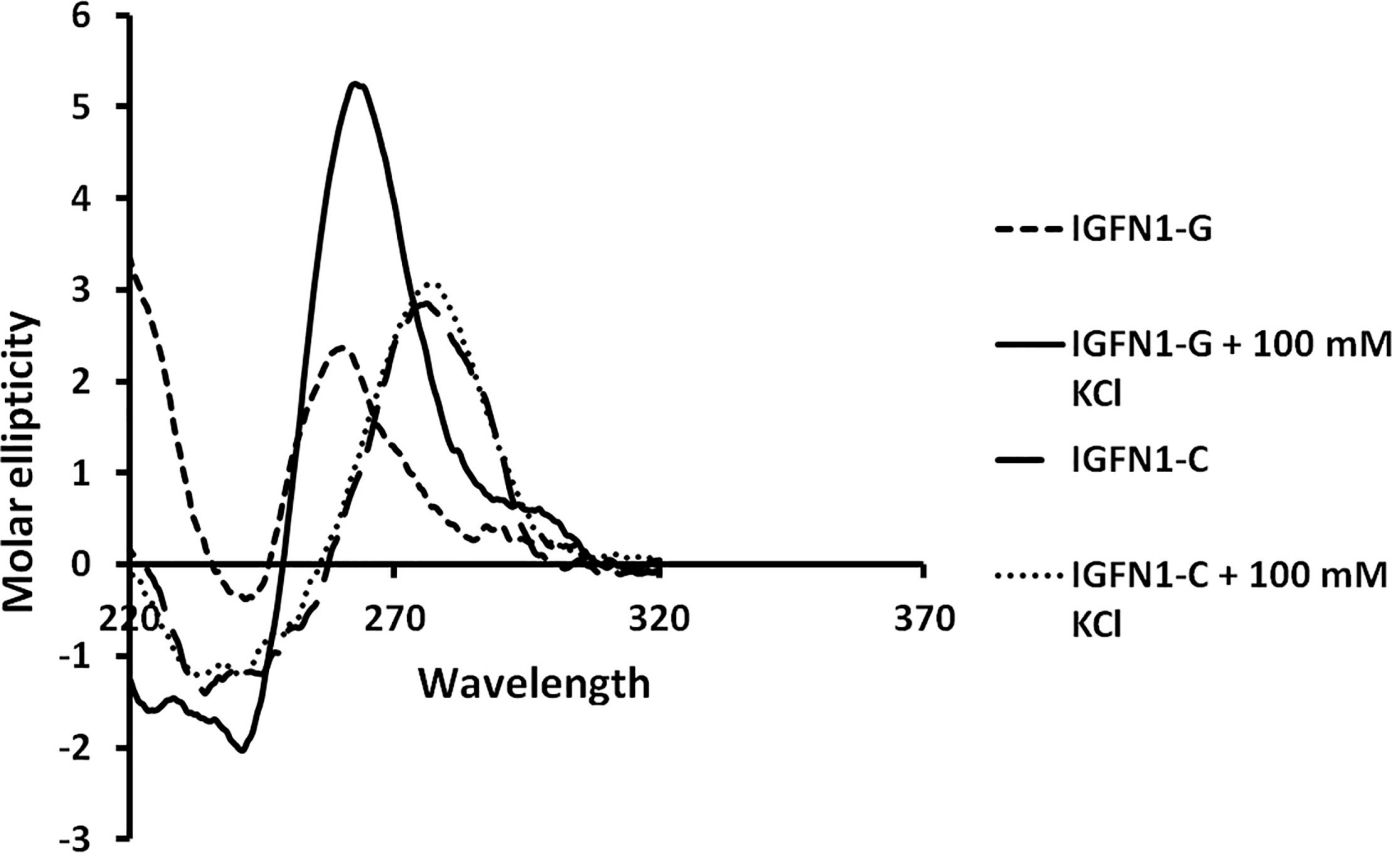
CD spectroscopy. Secondary structure study by circular dichroism showing the formation of G-quadruplex in the IGFN1-G and IGFN1-C in presence and absence of 100 mM KCl.

Characteristic G-quadruplex structures were displayed by CD spectroscopic study. CD spectra of IGFN1-G & C sequences in presence and absence of 100 mM KCl were captured. Molar ellipticity a measure of G-quadruplex structure increases with the addition of KCl in IGFN1-G sequence whereas addition of KCL has no effect on IGFN1-C oligo. CD spectra was analysed which displays the characteristic signature of G-quadruplex, showing a negative peak at 240 nm and a positive peak at 263 nm a characteristic feature of parallel G-quadruplexes (Fig 4).

### 5. PQS of IGFN1 intron 15 preferentially stops Taq DNA polymerase

**Fig 5:**
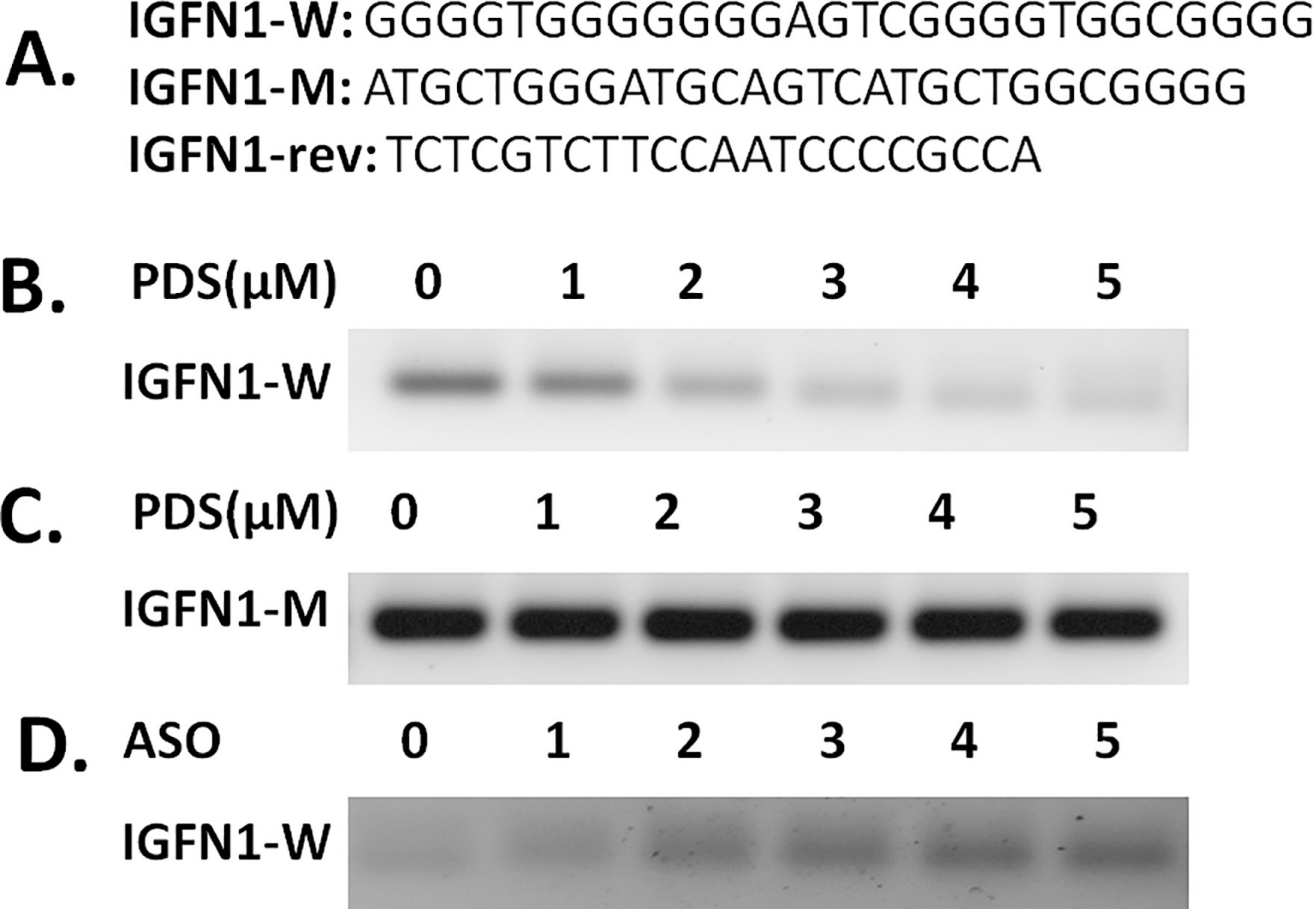
PCR stop assay. Oligo sequence of IGFN1 wild type(IGFN1-W), mutant type(IGFN1-M) and partially complementary(IGFN1-rev) oligo used in PCR stop assay, overlapping complementary last G-repeat sequence is underlined (A); Gel picture showing the inhibition of Taq DNA polymerase extension leading to the PCR stop at 0, 1, 2, 3, 4, 5 μM PDS concentration in IGFN1-W (B) and IGFN1-M oligo (C); PCR stoppage by G-quadruplex stabilization in presence of 4 μM PDS was rescued in presence of different molar concentration (0, 1, 2, 3, 4, 5 times) of IGFN1-C antisense oligonucleotide (ASO) with respect to molar concentration of IGFN1-W (D).

Predicted PQS was studied by PCR stop assay to study the effect of stable G-quadruplex structure on *Taq* DNA polymerase during DNA amplification. IGFN1 intron 15 G-quadruplex stability was investigated in presence of PDS and ASO. IGFN1-W DNA oligomer stabilized into G-quadruplex structure and blocks the partial hybridization with an complimentary oligo (IGFN1-rev) having partial complementarity to the last G-repeat leading to the inhibition of 5’ to 3’ extension with *Taq* DNA polymerase. With addition and increasing concentration of PDS (0, 1, 2, 3, 4, 5μM) IGFN1-Wild oligo formed the stable G-quadruplex structures and the stability increased with increasing concentration of PDS, inhibiting the double stranded PCR product. IGFN1-Mutant oligo is unable to form stable G-quadruplex because only one G stretch at 3’ end is present others are mutated leading to the partial hybridization of complementary oligo and PCR extension. Antisense oligo IGFN1-C hybridize with the IGFN1-Wild oligo and occupy the space of PDS binding and G-quadruplex formation hence acts as inhibitor of the G-quadruplex formation. With increasing concentration of IGFN1C, G-quadruplex formation is inhibited leading to the PCR extension (Fig 5).

### 6. Mapping of G-quadruplex

**Fig 6:**
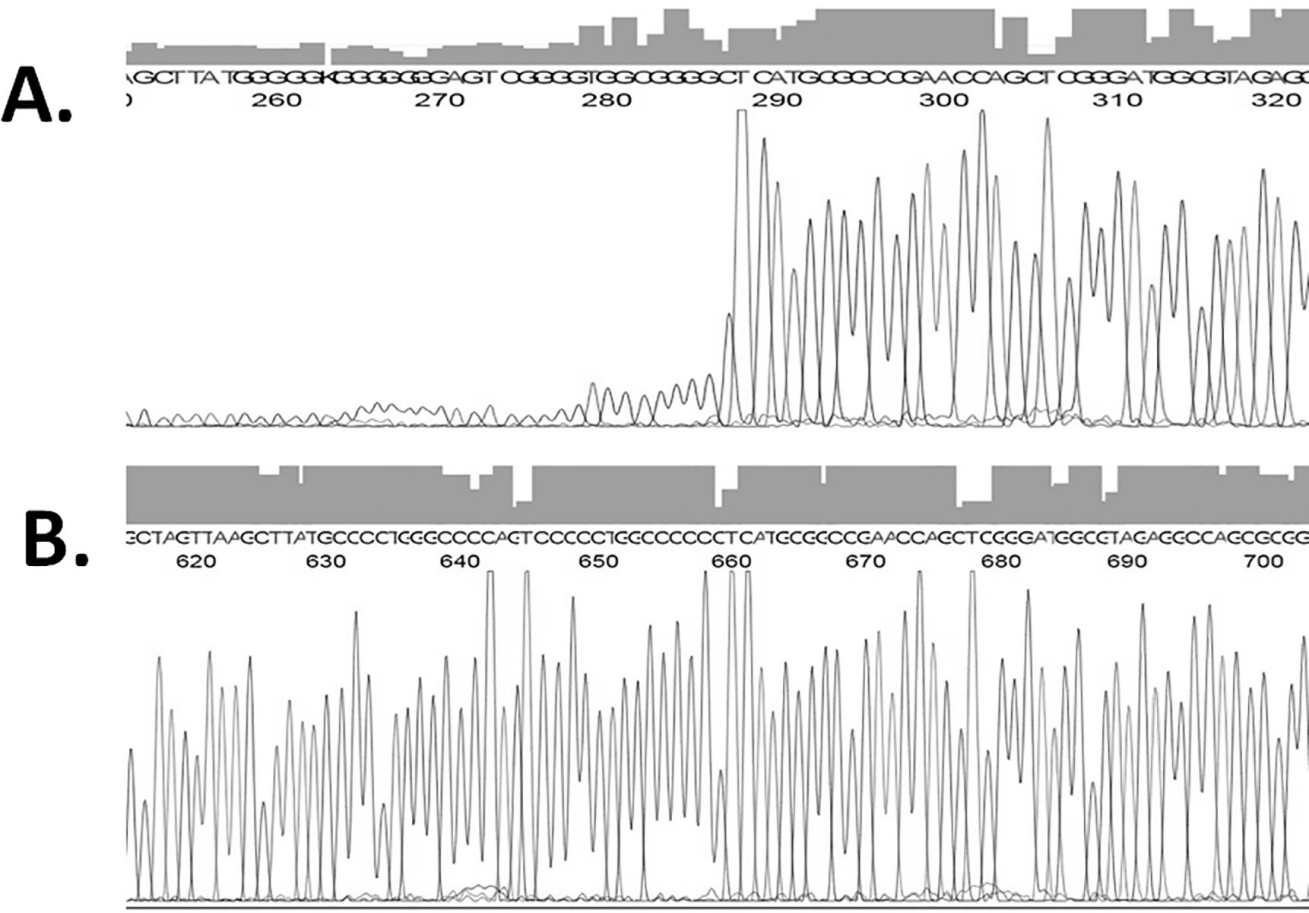
Mapping of G-quadruplex site by Sanger sequencing. Sequencing of IGFN1-wild (A) and IGFN1-mutant (B) plasmid in presence of 2 μM PDS.

A novel approach was developed and applied to exactly map the site of G-quadruplex formation with nucleotide level resolution using the automated Sanger sequencing. With addition of 0, 1, 2, 3, 4, 5 μM PDS in IGFN1-Wild plasmid sequencing, G-quadruplex stabilization inhibits the DNA sequencing polymerase activity by forming a highly stable DNA structure at the PQS start site confirming a stable G-quadruplex structure. Due to inhibition of polymerase activity, peak intensity significantly reduces at and from the start of PQS. In case of IGFN1-Mutant plasmid PQS was mutated hence unable to form the G-quadruplex on the template strand even in presence of PDS resulting no reduction of peak intensity (Fig 6).

### 7. RNA G-quadruplex block reverse transcriptase elongation

**Fig 7:**
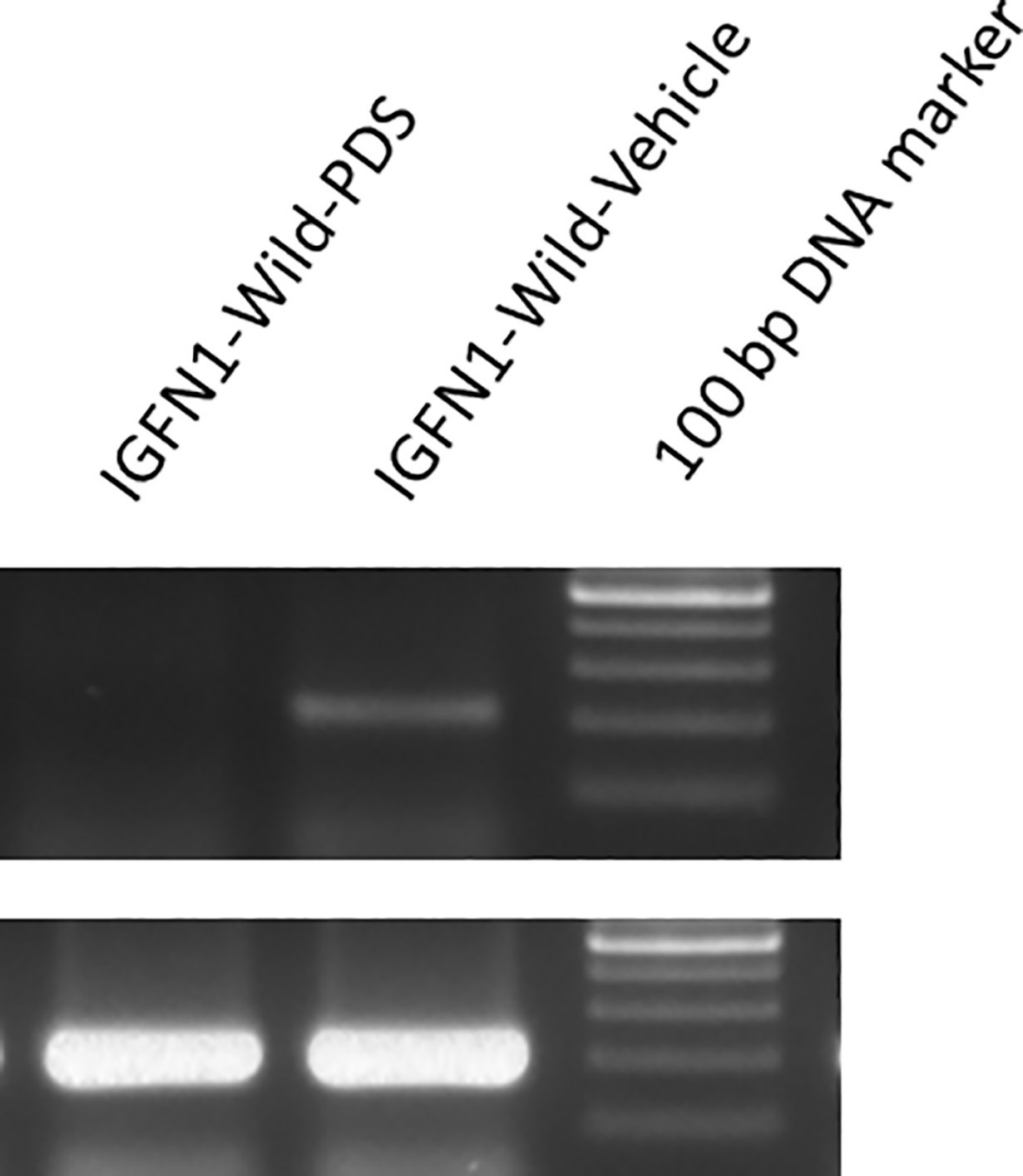
PDS stops reverse transcriptase elongation leading to the inhibition of RT-PCR amplification. Upper panel shows the RT-PCR amplification inhibition by PDS whereas this inhibition is absent in control. Lower panel shows the amplification of GAPDH, no difference in amplification is observed between PDS and control.

Stable G-quadruplex formation in RNA molecule and its effect on reverse transcriptase was studied according to Katsuda et al. 2016 with some modification. COS-7 cells were transfected with IGFN1-Wild plasmid and total cellular RNA was isolated. cDNA synthesis was performed in presence of 3μM PDS. RT-PCR was performed from this cDNA using the upstream and downstream to the IGFN1 PQS. Amplification was observed in water control whereas no amplification was observed in PDS treated cDNA. This shows the stoppage of reverse transcription during the cDNA synthesis of RNA by PDS. No difference in PCR amplification of GAPDH was observed. During cDNA synthesis, PDS stabilizes G-quadruplex in RNA molecule and inhibits or stops the cDNA synthesis on RNA molecule leading to the inhibition of RT-PCR amplification (Fig 7).

### 8. PDS enhances the splicing efficiency of IGFN1 intron 15 in UOK146 cell line

**Fig 8:**
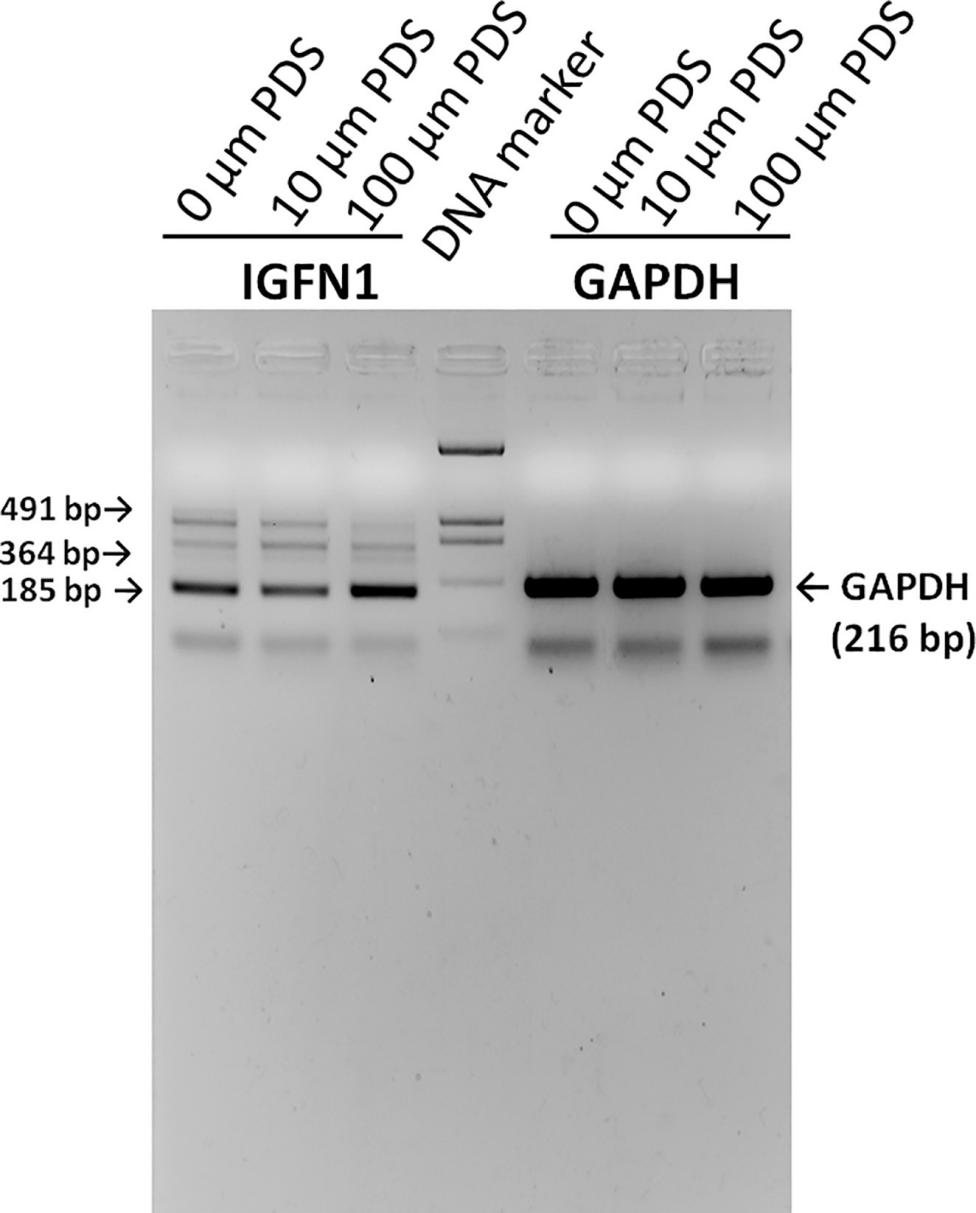
Role of PDS in splicing of IGFN1 intron 15. RT-PCR from the cDNA of UOK146 cell line after the treatment with 0, 10, 100 μM PDS for 24 hrs.

As mentioned earlier IGFN1 intron 15 spliced out in 3 types of events, complete splicing, 5’ exon extension and a novel exon. Three different alternative splicing products namely A, B and C were formed by the differential use of intron 15. Role of G-quadruplex in the differential intron use was studied by the treatment of UOK146 cell line by PDS (10, 50, 100 μM) followed by RT-PCR. Relative amount of all the three different isoform changed after treatment with highest expression of complete intron splice product. This assay shows the involvement of G-quadruplex in the alternative splicing of IGFN1 in UOK146 cell line (Fig 8).

## Discussion

Many diseases, particularly some neurodegenerative disorders are proven to be caused by G-quadruplex sequences [26,12,13]. This non-B DNA is also studied for its involvement in many types of cancers e.g. stomach, liver T-cell leukemia and follicular lymphoma etc. [10,18,19,27]. Intronic G-quadruplex affect the splicing of many genes such as hTERT, FMRP, TP53, PAX9, BACE2 [21, 28–31]. Theses splicing events lead to the enrichment of pathogenic form or increases the activity of particular isoform which is the reason behind the disease pathogenesis. In RCC novel splicing variants were identified in IGFN1 transcripts and validated by Sanger sequencing. Two novel variants one with 5’ exon extension and other with a novel exon were identified. Novel exon and the 5’exon extension in the IGFN1 has significant role in the relative expression of different isoforms of IGFN1. Identification of the factors and features which could be used or targeted to change the relative expression could be of therapeutic importance. Since two novel isoforms have been discovered in the present study, the role of these novel isoform towards cancer more particularly RCC could be of interest for biomarker discovery or therapeutics intervention.

G-quadruplex sequence in the region near splicing alteration has impact on the splicing, therefore it has been explored to know the effect of G-quadruplex on IGFN1 splicing. A PQS with high G-score has been predicted in intron 15 of IGFN1 mRNA. Folding of this PQS into G-quadruplex was studied by gel shift assay and many intra and intermolecular G-quadruplexes with increased and decreased mobility was observed. Its stability and involvement in biological events has been studied by PCR stop assay. PDS stabilized the formation of G-quadruplex in the IGFN1 G-quadruplex in wild type oligo and inhibited the PCR extension of primer and this PCR inhibition was concentration dependent. PCR amplification with IGFN1-mutant oligo has no effect with addition of PDS or increasing its concentration. Addition of ASO rescued the PCR inhibition caused by PDS in ASO in concentration dependent manner. Secondary structure formation of this PQS at biophysical level was studied by CD spectroscopy. IGFN1-G oligo with addition of KCl stabilizes into G-quadruplex and this stabilization abolished in IGFN1-C oligo. Pyridostatin (PDS) was reported to be a G-quadruplex stabilizer and investigated for its behaviour and it has selectivity towards interaction with G-quadruplex [32]. In present work we provide evidence from *in vitro* and cellular studies that PDS interacts with the PQS and stabilize into G-quadruplex. Detection of G-quadruplex formed by PQS was performed by automated Sanger sequencing approach and inhibition of peak intensity was observed exactly at the first base of the PQS. IGFN1-Mutant plasmid has C in place of G, thus lost the ability to form the G-quadruplex hence no peak intensity inhibition was observed. This study shows for the first time the use of automated Sanger sequencing for the detection and confirmation of G-quadruplex at single base resolution. This is a sensitive specific approach and can be applied for the screening of G-quadruplex stabilizer and inhibitor. These G-quadruplexes are stable in cellular condition and affect biological processes. Effect of IGFN1 G-quadruplex in cellular condition in alternative splicing was studied by the treatment of PDS followed by expression study at mRNA level by RT-PCR. IGFN1 isoform expression varies with PDS treatment and with changing the concentration in UOK146 cell line in which this alternative splicing was discovered. This study shows that IGFN1 alternative splicing changes with the treatment with PDS in UOK146 cell line, therefore IGFN1 splicing could be targeted as therapeutics in cancer and other diseases. In conclusion, present study provides evidences of novel splicing alteration in IGFN1 and a stable G-quadruplex formation in IGFN1 intron 15 is involved in splicing efficiency and expression of different isoforms of IGFN1.

Studies have shown that G-rich region/G-quadruplexes are involved in the splicing regulation. β-Tropomyosin intron 6 has 6 repetitions of (A/U)GGG motif acting as splicing enhancer and mutations in these motifs decreases(≈75%) splicing efficiency[33]. The Intron 2 of human alpha-globin gene contains many G triplets near 5’ end and mutations in these triplets reduced splicing efficiency *in vivo* [34]. Human PAX9 intron 1 have 11 repeats of (A/U)GGG motif and if guanines were changed by adenines, expression of spliced PAX9 intron 1 decreased dramatically. Treatment with 360A, a ligand which selectively binds with G-quadruplex increased the splicing efficiency even further (60%) [30]. As discussed above, genes having G-rich/G-quadruplex sequences has role in splicing regulation. Therefore isoform specific gene expression could be regulated by different chemicals/drugs like 360A, PDS and TmPyP4 having the property to interact with G-quadruplex and stabilize it. These G-quadruplexes are now considered for the drug designing and therapeutic medicines for the disease treatment [35–37].

In conclusion the results provide evidence of novel splicing in intron 15 of IGFN1 leading to the formation of two more alternative spliced isoforms. PQS in this intron forms a stable G-quadruplex which is involved in the alternative splicing and regulates splicing efficiency. Since biological role of IGFN1 is isoform dependent therefore G-quadruplex study lead to better understanding of its expression and could be targeted for therapeutic intervention.

## Materials and methods

### RNA isolation, cDNA synthesis and RT-PCR

Total cellular RNA from the cultured cells was purified using the Trizol reagent (Sigma, USA) and treated with DNaseI to remove any contaminating DNA. After purification 2 μg of this DNA free RNA was taken for cDNA synthesis using High Capacity cDNA Reverse Transcription kit (Thermo Scientific, USA) according to the manufacturer’s instructions.

### Potential G-quadruplex sequence (PQS) prediction

IGFN1intron 15 sequence was retrieved from NCBI and analysed by online tools such as a prediction web-based server QGRS Mapper [38]. Parameters for QGRS Mapper were: Max length: 30; Min G-group: 3; loop size: 0 to 5 [39]. IGFN1intron 15 sequence was also analysed by a database of predicted non-B DNA-forming motifs *nonB-DB* which integrates annotations and enables analysis of nucleic acid sequence for non-B DNA structure formation. Non-B DNA motif search tool (nBMST) of non-B DB was used to analyze the DNA sequence for G-quadruplex forming repeat search [40].

### Gel shift assay

Formation of G-quadruplex was confirmed by by gel shift assay by nondenaturing PAGE. IGFN1-G and IGFN1-C oligos were mixed with 100 mM KCl and NaCl and incubated at 37˚C for 1 hr and then resolved on 15% native PAGE at 120 V and 4˚C for 15 hrs. Gel was stained with 0.5 μg/ml Ethidium Bromide solution for 30 minutes and destained for 30 minutes and visualized in UV light.

### Circular dichroism

IGFN1-G and IGFN1-C oligonucleotides at 5 μM concentration were incubated with 100 mM KCl in 10 mM Tris HCl, pH 7.4 at 37°C for 1hr. Circular Dichroism spectra was recorded on a JASCO J-815 spectrometer. Three scans were performed at 20°C from 220 to 320 nm with the buffer spectrum subtraction [41].

### Plasmid constructs

IGFN1PQS was cloned in pcDNA3.1myc expression vector upstream to the TFE3 cDNA sequence (IGFN1-Wild). Underlined G bases (Fig 2) are mutated to C to form the IGFN1-Mutant plasmid. G-quadruplex will form in sense strand in IGFN1-Wild type plasmid and in antisense strand in mutant type plasmid.

### Mapping of G-quadruplex by Sanger sequencing

IGFN1-Wild and Mutant plasmids were used for cycle sequencing in presence of 0, 1, 2, 3, 4, 5 μM PDS with the primer and ABI big dye terminator kit following manufacturer’s instructions. Reaction products were purified by ethanol and Sodium Acetate precipitation and sequenced on ABI 3130 genetic analyzer [42].

### PCR stop assay

G-quadruplex structure stabilization by PDS and inhibition by Antisense Oligonucleotide (ASO) was studied by PCR stop assay. In this assay either wild type oligonucleotide (IGFN1-W) having G-quadruplex or its mutant (IGFN1-M) was used with a partially complementary oligonucleotide (IGFN1-rev) that partially hybridizes to the last G-repeat of the wild and mutant type oligos (Fig 2). The chain extension reaction was performed in 1× PCR buffer containing 0.05 mmol/L dNTPs, 3 units Taq DNA polymerase, 20 pmol oligonucleotides with different concentrations of PDS. PCR conditions: 94°C for 2 min followed by 30 cycles of 94°C for 30 s, 58°C for 30 s, and 72°C for 30 s. Amplified PCR products were resolved on 3% Agarose gels in 1× TAE buffer and stained with ethidium bromide[28].

### Cell lines, and transfection

Cells were cultured in DMEM supplemented with 10% fetal bovine serum and 100 U/ml penicillin, 100 μg/ml streptomycin antibiotics and grown in 5% (v/v) CO2 in a humidified incubator. 70–90% confluent cells were transfected in 6-well plate by Lipofectamine 2000(Life Technologies) according to manufacturer’s instruction.

## Supporting information

S1 File Sanger sequencing of three isoforms of IGFN1. (DOC)
