# Supplementary material for: Novel splicing in IGFN1 intron 15 and role of stable G-quadruplex in the regulation of splicing in renal cell carcinoma
Source: PLoS One. 2018 Oct 18;13(10):e0205660. doi: 10.1371/journal.pone.0205660 (PMC6193652; doi:10.1371/journal.pone.0205660)
Supplement: S1 File — (DOC) [file pone.0205660.s001.doc]

**Sanger sequencing of three isoforms of IGFN1:**

**IGFN1Exon 15**

**IGFN1Intron 15**

**IGFN1Exon 16**

ATTCCCCTACCATTGCTCCAGATGTGACAGAGAAACTGAGAGAGCCACTGGTGGTCAAGGCTGGGAAGCC

GGTGATAGTGAAGATCCCCTTCCAGAGCCACCTCCCCATTCAGGCTGCCTGGAGGAAGGACGGGGCTGAG

GTGGTGGGCAGCAGTGACAGGGAGGCCCAGGTGGACCTGGGGGATGGCTACACGCGGCTGTGCCTCCCCA

GCGCAGGCAGGAAGGACTGTGGCCAGTACAGCGTGACACTGAGGAGTGAGGGAGGCTCTGTGCAGGCCGA

GCTCACTCTGCAAGTCATAG

GTACCAGCCCTGTCTTCCCCCAACTAAGGCCTGAGAGTCCCTGGGGTTCTGGGCCCTCCCTGCCATCAAG

GGCAGGCCTGGGATATGATCTCTGCTGGCTCTGATCCTCATTTGCATGCTGTTTAAG**ATCCAGACCT**

**ACCACTAACTCTGCTCAGACTGCTTAGGAAGAAGCAGTTGAGATGAACACAGT**

**GCTGGGCTCCTGCTGGGGGGGAGGAGCCGGTTATGCTTCTCTGACCCCAATAC**

**ACTGGGCCCTCAGGAAGTTGCTGGGTAGGATGGTGGTCCCCAGCCCTGAGTGG**

**TGCCAGCAAG**GTAGGACTGCTCTTGGCCGAGCAGGTGCCCATACCAGCCACCGGACGGTGGGGCCGGG

AGGGGCTGGTGCATTCCATGGCACATCCCTGGGTGCATGTGTGTGTTGGGGGTGGAGACATCTCAAGAATG

TGTGGCCCTCTGGGTAATGCCCAAGGCTCTCTGGACAGTCTGCCCCATTGCAGGCTTATCTGCTGATGAGT

GGATG**GGGG**TGGG**GGGG**AGTC**GGGG**TGGC**GGGG**AGTTGGGGGGGTTGGCGCTGCTCCCCTCCAGCT

CCTCTGACCCCTCCTCTTCCCGCTCCTCTCTGTGGGTCCCCAG

ACAAGCCTGATCCCCCACAAGGCCCCATGGAGGTTCAGGATTGCCATAGGGCTGGCGTCTGCCTCCGCTG

GCGGCCCCCAAGGGACAATGGGGGCCGGACTGTAGAGTGCTACGTGGTGGAGAGACGGCAGGCTGGCAGG

AGCACTTGGCTGAAGGTGGGCGAGGCCCCCGCTGACAGCACCACCTTCACGGATGCCCATGTGGAGCCAG

GCAGGAAGTATACCTTCCGAGTGCGGGCTGTGACCTCAGAGGGGGCTGGCGAGGCCCTGGAGTCTGAGGA

GATATTGGTGGCTCCTGAGG

**IGFN1-A**

AKATTGTGACAYTGAGGAGTGAGGGAGGCTCTGTGCAGGCCGAGCTCACTYTGCAAGTCATAGACAAGCCTGATCCCCCACAAGGCCCCATGGAGGTTCAGGATTGCCATAGGGCTGGCGTCTGCTCCGCTGGCGGCCCCCAMAARGGRRMAAKWKGWGGT

**IGFN1-B**

TTYTGACACTGAGAGTGAGGGAGGCTCTGTGCAGGCCGAGCTCACTCTGCAAGTCATAG**ATCCAGACCTACCACTAACTCTGCTCAGACTGCTTAGGAAGAAGCAGTTGAGATGAACACAGTGCTGGGCTCCTGCTGGGGGGGAGGAGCCGGTTATGCTTCTCTGACCCCAATACACTGGGCCCTCAGGAAGTTGCTGGGTAGGATGGTGGTCCCCAGCCCTGAGTGGTGCCAGCAAG**ACAAGCCTGATCCCCCACAAGGCCCCATGGAGGTTCAGGATTGCCATAGGGCTGGCGTCTGCCTCCGCTGGCGGCCCCCAAGGACAATKGTCT

**IGFN1-C**

CYTKGKKKMGGCSAARCYYCTCTGCAGTCATAGGTACCAGCCCTGTCTTCCCCCAACTAAGGCCTGAGAGTCCCTGGGGTTCTGGGCCCTCCCTGCCATCAAGGGCAGGCCTGGGATATGATCTCTGCTGGCTCTGATCCTCATTTGCATGCTGTTTAAR**ATCCAGACCTACCACTAACTCTGCTCAGACTGCTTAGGAAGAAGCAGTTGAGATGAACACAGTGCTGGGCTCCTGCTGGGGGGGAGGAGCCGGTTATGCTTCTCTGACCCCAATACACTGGGCCCTCAGGAAGTTGCTGGGTAGGATGGTGGTCCCCASCCCTGAGTGGTGCCAGCAAG**ACAASCCTGATCCCCCACAAGGCCCCATGGAGGTTCAGGATTGCCATAGGGCTGGCGTCTGCCTCCSCTGGCGGCCCCCAAGGGACAATGGGGGCCGGACTGTWGAGTGCTACGTGGTGRA
